# Gallium-Containing Materials and Their Potential within New-Generation Titanium Alloys for Biomedical Applications

**DOI:** 10.3390/biomimetics8080573

**Published:** 2023-11-30

**Authors:** Rhianna McHendrie, Wenlong Xiao, Vi Khanh Truong, Reza Hashemi

**Affiliations:** 1 College of Science and Engineering, Flinders University, Adelaide, SA 5042, Australia; mche0012@flinders.edu.au; 2 School of Materials Science and Engineering, Beihang University, Beijing 100191, China; 09641@buaa.edu.cn; 3 College of Medicine and Public Health, Flinders University, Adelaide, SA 5042, Australia; vikhanh.truong@flinders.edu.au

**Keywords:** gallium, titanium alloys, antibacterial biomaterials, medical implants

## Abstract

With the rising demand for implantable orthopaedic medical devices and the dominance of device-associated infections, extensive research into the development of novel materials has been prompted. Among these, new-generation titanium alloys with biocompatible elements and improved stiffness levels have received much attention. Furthermore, the development of titanium-based materials that can impart antibacterial function has demonstrated promising results, where gallium has exhibited superior antimicrobial action. This has been evidenced by the addition of gallium to various biomaterials including titanium alloys. Therefore, this paper aims to review the antibacterial activity of gallium when incorporated into biomedical materials, with a focus on titanium-based alloys. First, discussion into the development of new-generation Ti alloys that possess biocompatible elements and reduced Young’s moduli is presented. This includes a brief review of the influence of alloying elements, processing techniques and the resulting biocompatibilities of the materials found in the literature. The antibacterial effect of gallium added to various materials, including bioglasses, liquid metals, and bioceramics, is then reviewed and discussed. Finally, a key focus is given to the incorporation of gallium into titanium systems for which the inherent mechanical, biocompatible, and antibacterial effects are reviewed and discussed in more detail, leading to suggestions and directions for further research in this area.

## 1. Introduction

The increasing number of implantable medical devices such as orthopaedic implants, owed partly to the prevalence of related health problems and an aging population, has led to an amplified incidence of device-associated infections [1]. The development of biofilm formation on the surfaces of implants can result in chronic implant-related infections, and hence, implant failure [1]. Despite healthy and efficient host immune systems, bacteria can quickly colonise the implant surface, leading to persistent infections, implant failure, and potentially significant complications. The need for antibacterial implant materials is therefore imperative. The development of implant materials with inherent antibacterial properties is crucial to mitigate the risks associated with bacterial colonization on implant surfaces, ensuring the success of the implant, preventing infections, and upholding the well-being of patients. Having attracted extensive attention as biomedical materials, Titanium (Ti) and its alloys depict a nominal Young’s modulus, great strength, and good biocompatibility and corrosion properties [2,3,4,5]. This desirable combination of properties has rendered titanium alloys one of the most suitable biometals for the manufacture of orthopaedic implants such as total hip replacement systems [6,7,8]; however, titanium alloys do not generally offer a great level of resistance against tribocorrosion [9,10,11]. The clinical benchmark among titanium alloys for orthopaedic implant applications is the α/β alloy Ti-6Al-4V. However, the disproportionate Young’s modulus between Ti-6Al-4V (110 GPa) compared to cortical bone (5–30 GPa) can result in stress-shielding effects, which may instigate implant failure and bone resorption in large, permanent hip joint implants [4,12,13]. Moreover, it is well reported that aluminium (Al) and vanadium (V) can be released from the alloy to elicit serious cytotoxic effects, including neurodivergent diseases and genetic damage [14,15]. These details have motivated inquiries into Ti-based alloys with a Young’s modulus closer to bone, paired with biocompatible alloying elements for biomedical implant applications. 

Novel β-type Ti alloys with non-toxic alloying elements and reduced stiffness levels (Young’s moduli) have been the focus of extensive research development in recent years. Among the developed systems, Ti-Nb and its alloys have garnered interest as a result of their improved properties, including desired phase stability, lower Young’s modulus, and excellent biocompatibility and corrosion resistance [12,16]. Despite these properties, the major limitations of Ti alloys include the prospect of bacterial infection after implantation, and in some cases, their poor biocompatibility [4]. By controlling thermo-mechanical treatments and via selective alloying, biomechanical and biocompatible properties can be tuned. In addition, antibacterial effects have been demonstrated through the addition of metallic elements such as gallium (Ga). Ga prevents implant biofilm formation and provides antibacterial activity through a mechanism replacing Iron (Fe) nutrients within bacterial metabolism [17]. This has allowed Ga to be used in the development of biomaterials with antibacterial properties, where even small additions of Ga (1–2 wt%) decreased the biofilm viability of the bacterial strain *S. aureus* by approximately 50% in vitro [18,19]. The addition of Ga to low-stiffness Ti-alloys may therefore promote the development of a new class of biometallic alloys that can offer similar properties to bone, and that also exhibit antibacterial activity.

This review therefore aims to assess the development of Ti-alloys comprising biocompatible elements with low stiffness levels by examining their mechanical and biological compatibilities for orthopaedic implant applications. Additionally, it aims to explore the antibacterial effects reported for gallium-based material systems. Finally, this review analyses the incorporation of gallium into titanium-based systems and the imparted antibacterial activity, mechanical properties, and cytocompatibilities. This review also aims to provide a basis for future research directions on Ti-Ga based alloys that can beneficially offer antibacterial characteristics within orthopaedic implant applications.

## 2. β-Type Titanium Alloys with Biocompatible Elements

Numerous studies have been dedicated to the development of titanium alloys with a low Young’s modulus comparable to that of cortical bone (5–30 GPa) in addition to the incorporation of biocompatible alloying elements [2,4,20,21,22,23,24,25,26]. Titanium alloys incorporating elements such as Zirconium (Zr), Niobium (Nb), Tantalum (Ta), and Tin (Sn) have undergone extensive investigation owing to their superior biocompatibility (Table 1) and promising potential for use in medical applications. This section aims to provide a brief review of β-type titanium alloys with non-toxic alloying elements and reduced stiffness levels. It should be noted that a more comprehensive review is reserved for Section 3, and particularly Section 4, as it is the main scope of this review article.

### 2.1. Influence of Alloying Elements on Microstructure and Mechanical Properties

**Figure 1:**
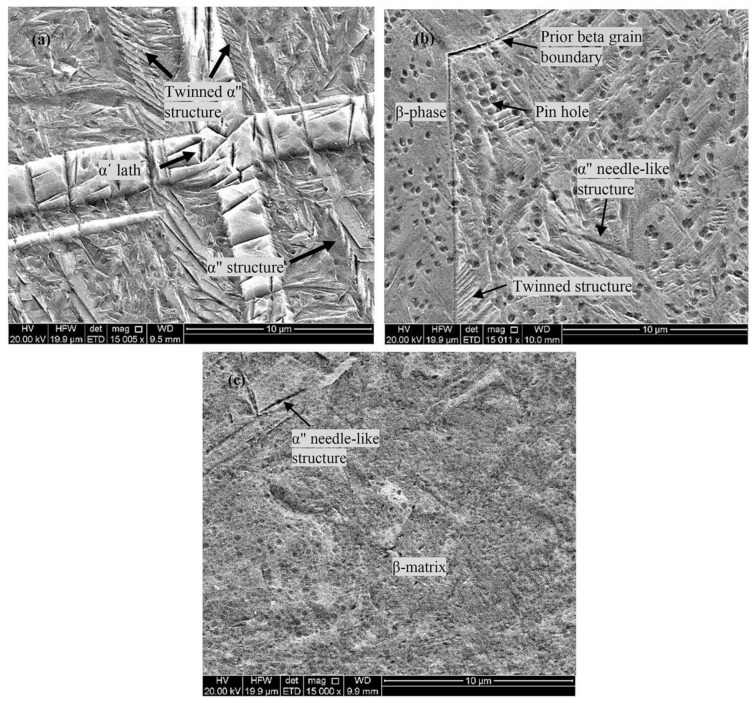
Scanning electron microscopy (SEM) images for (**a**) Ti-23Nb-7Zr showing α′ + α″ phase, (**b**) Ti-28Nb-7Zr showing α″ + β phase, and (**c**) Ti-33Nb-7Zr showing β + α″ phase at 15,000× magnification [32].

In recent years, significant progress has been made in developing titanium alloy compositions with improved mechanical properties that help resolve the limitations of stress-shielding, and that possess biocompatible elements [13,28,29,30]. New-generation β-type Ti alloys with biocompatible elements and body-centered cubic (BCC) crystal structures have been the focus of recent developments, as they possess reduced stiffness levels (Young’s moduli), which is desirable for implants in contact with bone, and improved wear resistance compared to α and α-β Ti alloys. Among the developed systems, Ti-Nb and its alloys have garnered immense interest as a result of their improved properties, including desired phase stability, lower Young’s modulus (around 65 GPa), and excellent biocompatibility and corrosion resistance [12,16]. In a notable study, Kuroda et al. [31] investigated the mechanical properties of eight Ti-Nb alloys via tensile testing, compared to conventional Ti-Al alloys. The Ti-29Nb-13Ta-2Sn alloy possessed the lowest Young’s modulus, reported at approximately 46 GPa, where all Ti-Nb alloys also exhibited a reduced modulus compared to the Ti-Al based alloys. In further elucidating the desirable effect of niobium addition to titanium systems, Tan et al. [32] studied the effect of niobium content on Ti-XNb-7Zr (X = 23, 28, 33) wt% alloys from a microstructural and mechanical perspective. Employing X-ray diffraction analysis and microstructure characterisation, it was concluded that the addition of Nb to the alloy system stabilised the β-phase microstructure. As shown in Figure 1, the microstructure changes from α′ + α″ phase for the 23 wt% Nb, to α″ + β phase for the 28 wt% Nb alloy, and to β + α″ phase within the 33 wt% Nb alloy [32]. By means of nanoindentation methods, the Young’s modulus was found to be minimised in the 33Nb wt% alloy composition, with a reported value of 29 GPa. These results emphasise that increasing additions of Nb are advantageous to mechanical properties, where [16] further elucidated that the minimum modulus for binary Ti-Nb alloys is 65 GPa when Nb alloying additions are approximately 40 wt%. However, while improvements to the Young’s modulus were observed, the hardness was largest in the Ti-23Nb-7Zr alloy, owing to the sizable α′-phase.

**Figure 2:**
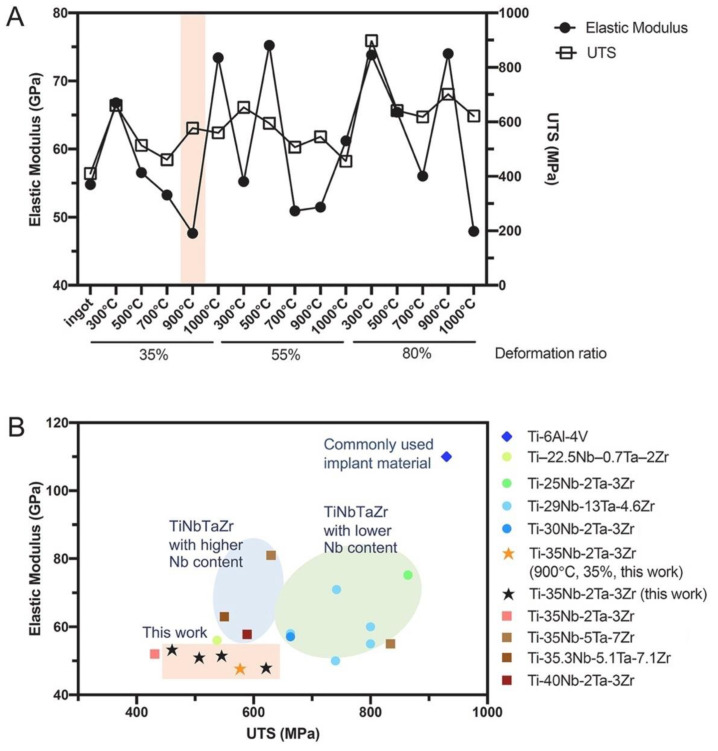
The mechanical properties of the developed Ti-35Nb-2Ta-3Zr alloy, with comparison to other literature alloys. (**A**) The elastic modulus and ultimate tensile strength (UTS) of the alloy (Ti-35Nb-2Ta-3Zr) when subjected to different processing techniques; (**B**) comparison of elastic modulus and UTS to other alloys developed in the literature [33].

Comparatively, using a combined approach, Mao et al. [33] developed mechanobiologically optimised Ti-35Nb-2Ta-3Zr alloys and analysed their mechanical properties using both conventional tensile tests and finite element models. The alloys exhibited low Young’s modulus values (47.6–53.3 GPa) and greater mechanobiological characteristics compared to conventional Ti-6Al-4V; however, only moderate strength (460–577 MPa) was observed within the combined methods [33]. A comparison of the developed alloys to other notable literature is depicted in Figure 2. While benefits to mechanical properties have been observed in the literature, a large dissimilarity between the Young’s modulus of β-type Ti-Nb alloys from that of bone still exists, which may instigate implant failure. This was observed in investigating the β-Ti-based Sn alloys Ti-32Nb-(2, 4)Sn [24]. Although a reduction in Young’s modulus (64–68 GPa) was observed with increasing Sn content, the modulus mismatch persisted, in addition to moderate strength.

While reduced stiffness has been demonstrated in the literature, only moderate strength and in some cases reduction in ductility are exhibited for β-Ti-Nb-type alloys compared to Ti-6Al-4V. To attain a minimal Young’s modulus, a good level of ductility, and a suitable level of strength, the role of interstitial elements such as oxygen (O) has been explored by Wang et al. [13]. In this work, the Ti-38Nb-0.5O alloy was developed and reported to exhibit a modulus of 52 ± 2 GPa, a ductility of approximately 26%, and an ultimate tensile strength of 1141 ± 17 MPa [13]. This study therefore highlighted the strengthening effect of oxygen on the Ti-Nb alloy [30,34]. Like oxygen, interstitial nitrogen (N) has also attracted attention due to its effect on strength and the martensitic transformation and deformation behaviour. For example, Ramarolahy et al. [35] investigated the influence of O and N incorporation to Ti-24Nb-0.5X (X = O, N) from both microstructural and mechanical perspectives. While XRD patterns reveal the presence of a β-phase microstructure for the alloys, it is noteworthy that a residual α″ martensite phase was observed in the Ti-24Nb-0.5N alloy [35]. This occurrence has the potential to detrimentally affect the material’s mechanical properties. Furthermore, in employing tensile tests, both Ti-24Nb-0.5O and Ti-24Nb-0.5N alloys depicted remarkably low stiffness levels, at 54 and 43 GPa, respectively. These alloys therefore exhibit Young’s moduli close to that of cortical bone (approximately 5–30 GPa). Despite the frequently observed decrease in ductility caused by interstitial elements, this study found that the inclusion of such alloying additions did not negatively affect ductility. Notably, the Ti-24Nb-0.5O alloy exceeded that of the Ti-6Al-4V alloy, with an elongation of rupture at 22% [35].

### 2.2. Influence of Processing Parameters on Mechanical Properties

In addition to the meticulous selection of alloying elements and the incorporation of interstitial elements, extensive research has been conducted into the role of processing techniques in optimising the mechanical properties of titanium alloy. It is widely recognised that a reduction in Young’s modulus can be observed by controlling thermomechanical treatments, and therefore, the phase transformations. Numerous research endeavours have investigated the effect of hardening mechanisms, including work hardening, grain boundary strengthening, or precipitation hardening [29,30,36,37,38,39,40,41,42]. Notably, the strength properties of β-Ti-Nb-based alloys can be increased drastically using work hardening methods, as evidenced by yield strengths reaching 900 MPa and ultimate tensile strengths within the range of 1000 MPa [30,36,37,38,39,43,44]. Matsumoto et al. [45] studied the influence of thermomechanical processing parameters on Ti-25.5Nb-9.4Sn, including quenching, cold rolling, and heat treatment. The alloy demonstrated an excellent Young’s modulus (49 GPa) comparable to that of cortical bone paired with high ultimate tensile strength (1017 MPa), and represents the enhancement of mechanical properties gained with selective thermomechanical treatment [45]. Contrarily, various authors have regarded work hardening to be ineffective in increasing the fatigue strength of β-type Ti-Nb-based alloys [36,38,43]. Another idealistic method of enhancing the strength without sacrificing stiffness is by employing grain boundary hardening. However, only moderate yield strength results have been obtained within the range of 300–500 MPa [46,47]. These conclusions therefore represent the need to carefully select the appropriate thermomechanical treatments such that mechanical properties can be optimised. Overall, the microstructural and mechanical analyses of new-generation Ti-alloys form the basis in informing the suitability of these alloys for use in orthopaedic implant applications; however, biocompatibility assays are necessary to assess their effects both in vivo and in vitro.

### 2.3. Influence of Alloying Elements on Biocompatibility

**Figure 3:**
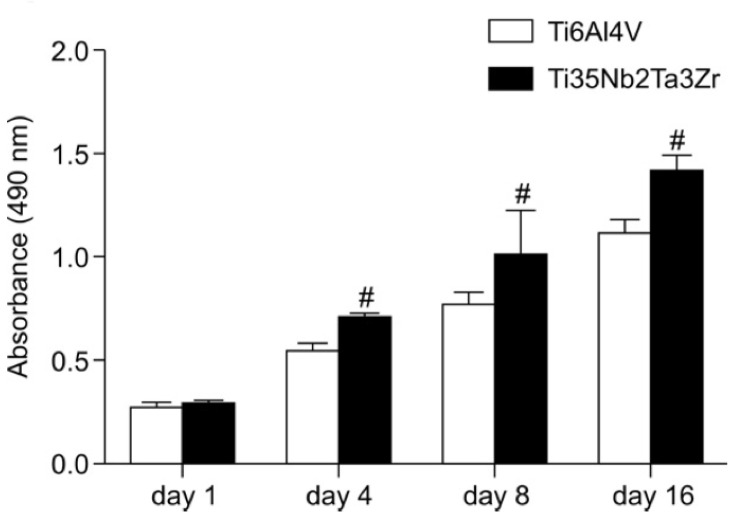
Osteoblast viability after 1, 4, 8 and 16 days on 88 disc samples of Ti-6Al-4V (control) and Ti-35Nb-2Ta-3Zr via an MTS assay. Data are expressed as mean ± standard deviation. # indicates *p* < 0.05 compared to Ti-6Al-4V [23].

The fabrication of novel Ti alloys with biocompatible elements and non-cytotoxic effects have been the focus of research in an attempt to extend the longevity of implants, and to improve their success after implantation. Analysis of the biocompatibility of orthopaedic Ti-alloys frequently encompasses assessments of their cytotoxicity, osseointegration, bone response, and corrosion resistance, in addition to analysing their mechanical properties and wear resistance. For example, Park [48] investigated the biocompatibility of Ti-13Nb-13Zr alloy using pre-osteoblastic cells and compared their attachment, spreading, viability, activity, and osteoblastic gene expression to those of Ti-6Al-4V. Compared to the control, the alloy depicted enriched properties including significantly increased cellular attachment and proliferation [48]. These favourable cell behaviour results were attributed to the use of non-cytotoxic alloying elements. Similarly, Guo et al. [23] studied the biological compatibility of the β-type titanium alloy Ti-35Nb-3Zr-2Ta via corrosion resistance tests and in vitro experiments to assess the spreading and proliferation of osteoblasts. Although the corrosion resistance was comparable to that of the measured Ti-6Al-4V alloy, osteoblast integration is believed to be greatly improved in the developed alloy compared to that of Ti-6Al-4V, as shown by the viability of osteoblast cells (cells responsible for the formation of new bones, as well as the growth and repair of existing bones) in Figure 3 for all samples. These results, paired with those of [20,49,50] indicate the promising application of these biomaterials for use as modern orthopaedic implants. Although Ti-Nb alloys have emerged as the focus of on-going research into metallic biomaterials, research into the cytocompatibility of promising elements such as Ga has been deficient.

## 3. Antibacterial Gallium-Based Materials

Gallium-based materials have emerged as promising antibacterial agents in inhibiting the activity of numerous bacterial strains, including both Gram-negative and Gram-positive bacteria. Among the developed materials, gallium has been incorporated into bioglasses, liquid metals, and bioceramics for its promising use in biomedical applications. For example, gallium nitrate employed in a phase 1 clinical trial has shown antibacterial effects against *P. aeruginosa* without any toxicity for cystic fibrosis patients, and therefore exhibits promising clinical antibacterial potential [51]. This section aims to review the incorporation of gallium into various materials with a focus on the imparted antibacterial properties.

### 3.1. Antimicrobial Mechanism of Gallium

The antimicrobial mechanism of gallium has been researched extensively [17,51,52,53] and is theorised to employ a “Trojan horse” strategy of bacterial inhibition. Gallium ions (Ga^3+^) possess an analogous ionic radius, electron affinity, and ionisation potential to that of ferric ions (Fe^3+^), and may therefore be mistaken for Fe^3+^ and bind strongly with iron-binding proteins involved in the metabolic and signalling processes of bacteria [17,51,54]. Siderophores are produced by bacterial cells and possess iron uptake systems, where Ga^3+^ competes with Fe^3+^ to bind to siderophores and essential enzymes and proteins [17]. Gallium is not redox-active, and when bound with iron-binding proteins can inhibit various iron-dependent redox pathways, and ultimately the function and subsistence of the bacterial cell [17,52,53]. Various studies have demonstrated gallium-to-siderophore binding ability. Rodriguez et al. [55] synthesised gallium complexed with the siderophore acinetoferrin, which demonstrated doubled antibacterial efficiency against the bacterial strain *M. tuberculosis*. Comparatively, Kelson et al. [56] established that large complexes such as Ga-staphyloferrin, Ga-cepaciachelin, and Ga-dihydroxybenzoyl-serine were significantly less effective at inhibiting bacterial activity compared to smaller complexes such as gallium citrate [56]. The authors clarified that the selection of appropriate siderophores is essential to the inhibitory mechanism of gallium. Although the siderophore-to-gallium binding ability has been demonstrated experimentally, studies outlined by [57,58,59] suggest that mutant strains of the bacteria *P. aeruginosa* can develop a resistance against simple gallium salts. It is therefore important to acknowledge that the mechanism of gallium activity and resistance is not comprehensively distinguished.

### 3.2. Gallium in Bioglasses

Scaffold materials that contain antibacterial elements have been applied as tissue engineering materials within the body due to their slow release of antibacterial agents. Existing gallium-based drugs often reach the maximum concentration of gallium (III) within a short time, resulting in a negligible antibacterial effect [60]. The sustained release of antibacterial elements is therefore critical to prevent iatrogenic infections, where biomaterials such as bioglasses have been employed to achieve slow release of antibacterial agents. Bioglasses, which are silicon-based glass–ceramic biomaterials infused with calcium and phosphorus, exhibit surface reactivity. When dissolved, they prompt the expression of osteogenic genes and angiogenesis. Conventionally, the synthesis of bioactive glasses involves melt-derived techniques, wherein the main network former, being silicate, borate, or phosphate, determines their classification as melt-derived glasses [60]. However, this method often incurs partial devitrification within crystalline phases [61]. This can lead to a reduction in biological properties, causing them to be unsuitable in producing scaffold materials, namely porous classes [61]. Because of these limitations, the sol-gel method has been regarded as a preferable alternative as it requires a lower processing temperatures and yields materials that exhibit higher specific surface area, nano porosity, and purity compared to their melt-derived counterparts [59,60].

**Figure 4:**
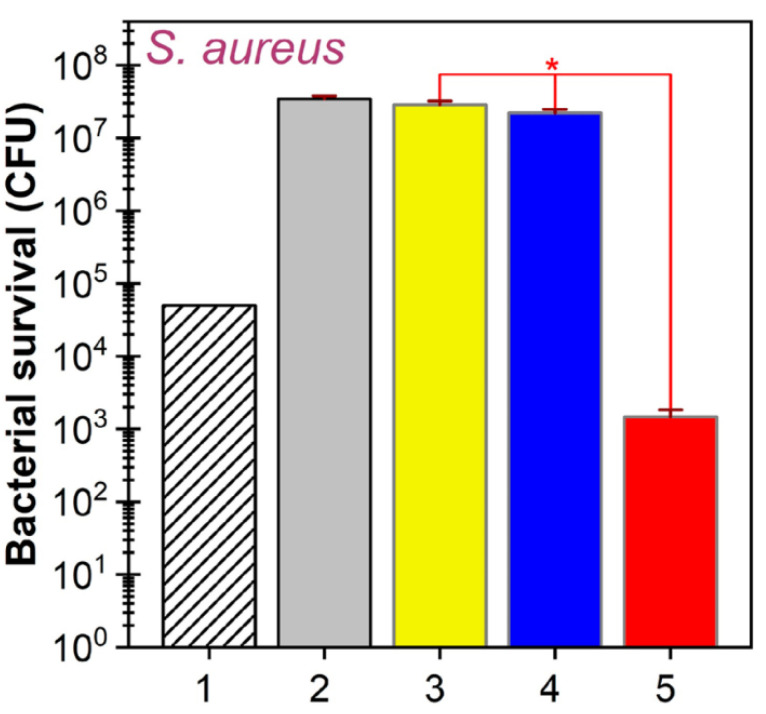
Bacterial survival of *S. aureus* after 24 h of (1) seeded CFUs; (2) control sample; (3) bare and silica-rich; (4) SiO2-CaO-P2O5-MgO-CaF2 sample; (5) Cu and Ga coated SiO2-CaO-P2O5-MgO-CaF2 substrate. * *p* < 0.05 [63].

Silicate-based bioglasses are frequently reported within the literature, where the addition of gallium has been demonstrated to impart enhancements to the structural and thermal properties of bioglasses, in addition to inducing antibacterial effects. Keenan et al. [62] investigated the antibacterial efficiency of bioglass Si_2_O-Na_2_O-CaO-ZnO using both broth dilution and agar disc diffusion methods. As much as 16 mol% Ga_2_O_3_ addition to the bioglass was monitored against bacterial strains of *E. coli*, *S. aureus*, and *C. albicans* [62]. The broth dilution method demonstrated that the addition of gallium to the sample depicted inhibition against *E. coli* and *C. albicans*, but no inhibition towards *S. aureus*. The agar disc diffusion study depicted an increased antibacterial effect compared to the broth dilution method. A significant reduction in biofilm bacteria was measured by the release of gallium ions from the addition of 3 wt% Ga_2_O_3_ to the matrix of SiO_2_-CaO-ZnO. Yielding similar antibacterial results but with a different sample type, Stan et al. [63] examined a number of bioglasses, with the best results observed for the Ga and Cu doped silica-based bioglass. Here, the Cu and Ga-coated SiO_2_-CaO-P_2_O_5_-MgO-CaF_2_ substrate depicted a decrease in magnitude of *S. aureus* by four orders after 24 h. This is shown in Figure 4, where the Cu and Ga bioglass sample reduced bacterial survival by 30 times compared to the seeded colony-forming unit (CFU), and by 4 orders of magnitude compared to the control samples. These outputs are promising for preventing biofilm formation at the implant surface.

**Figure 5:**
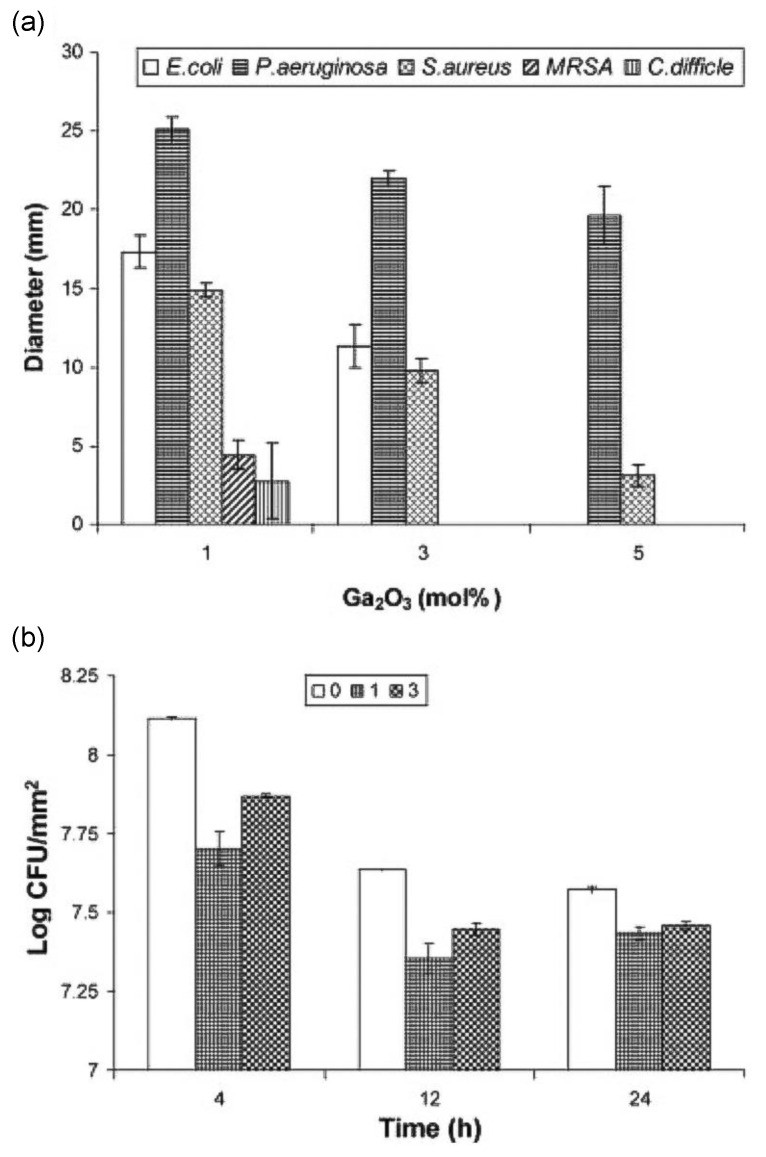
(**a**) Results of Ga_2_O_3_-doped phosphate-based glasses in 0, 1, 3 and 5 mol % using a disc diffusion assay against *S. aureus*, *E. coli*, *P. aeruginosa*, methicillin-resistant *S. aureus*, and C. difficile. (**b**) The viability of *P. aeruginosa* from 0, 1, and 3 mol % Ga_2_O_3_-doped phosphate-based glasses after 4, 12 and 24 h. Means were calculated from three specimens per sample type [67].

The demonstrated antimicrobial properties of gallium when incorporated into borate and phosphate glasses have also been demonstrated. The sustained and controlled release of gallium from a B_2_O_3_-Na_2_O-CaO-P_2_O_5_-ZnO glass system was observed over 28 days using the agar disc diffusion method [64]. No detectable inhibition was observed for the gallium-absent composition, while higher antibacterial effects against *P. aeruginosa* were observed with increasing concentrations of gallium [64]. Other studies [65,66] with comparable compositions have also demonstrated promising results in inhibiting bacteria when paired with gallium. By employing a different approach to study the action of Ga compared to [64], Valappil et al. [67] reported the effect of Ga-doped phosphate-based glass against five Gram-negative and Gram-positive bacteria strains via a disk diffusion assay. It was concluded that even 1 mol% of Ga addition was sufficient to cause a potent antibacterial effect by considerably inhibiting biofilm formation (Figure 5). This glass system therefore presents a favourable therapeutic agent alternative for pathogenic bacteria. Further evidence of inhibition against all these bacterial strains within phosphate glasses paired with gallium in 1 and 3 mol% has also been demonstrated in other studies [68,69].

**Figure 6:**
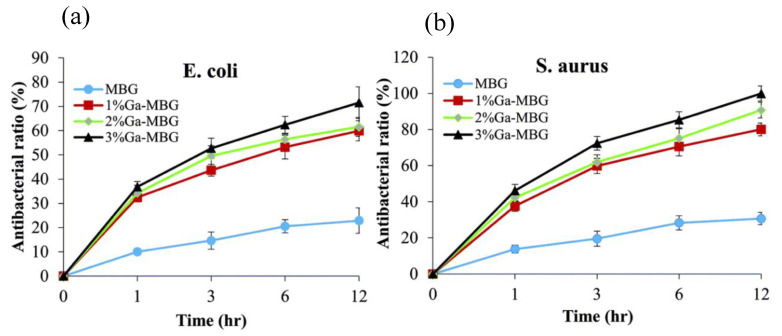
Antibacterial efficacy of Ga-doped mesoporous bioactive glasses (MBGs) against (**a**) *E. coli* and (**b**) *S. aureus* over 1, 3, 6 and 12 h [70].

In a recent study conducted by Pourshahrestani et al. [70], the antibacterial effect of sol-gel-derived 1–3 mol% Ga_2_O_3_ bioactive glasses 80SiO_2_-15CaO-5P_2_O_5_ (mol%) was investigated via an evaporation-induced self-assembly process. It was shown that the addition of 3 mol% Ga_2_O_3_ yielded the greatest antibacterial effect against the bacteria *S. aureus*, with 99% after 12 h of incubation, where all samples exerted an antibacterial effect against *E. coli* and *S. aureus* (Figure 6). Though the potent antibacterial effect of gallium-incorporated bioglasses against numerous bacterial strains has been reported, there exists evidence in the literature of Ga^3+^ being unable to be released from the glass network of bioglass samples to the surrounding medium. Studying the same sol-gel-derived bioglass matrix (80SiO_2_-15CaO-5P_2_O_5_) as [70], Salinas and Vallet-Regi [71] found that 3.5 mol% Ga_2_O_3_ substitution for SiO_2_ resulted in the negligible release of Ga^3+^ ions from the glass network. It was concluded that the sample therefore likely imparted no antibacterial property to the glasses. This demonstrates the necessity of further research into gallium’s antimicrobial mechanism, particularly within a range of samples.

### 3.3. Gallium in Liquid Metals

**Figure 7:**
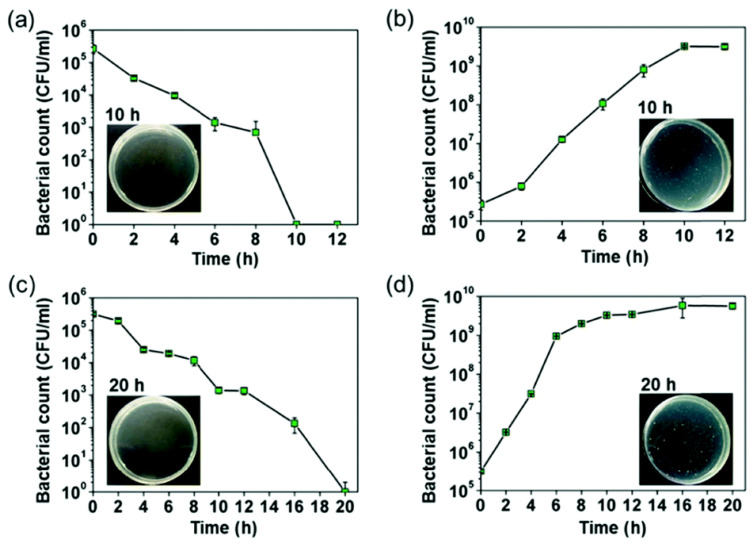
The growth of bacterial cells at varied incubation times: (**a**) *E. coli* on EGaIn viable cells; (**b**) *E. coli* viable cell count on PVC control; (**c**) *S. aureus* viable cell count on EGaIn; (**d**) *S. aureus* viable cell count on PVC control pieces [73].

Liquid metals (LMs) are elements or mixtures characterised by their state of liquidity at temperatures in close proximity to room temperature, and represent an emerging metallic material in research due to their excellent biocompatibility, metallic properties, and flexibility. LMs show attractive results when applied to biomedical therapeutics, owing to their tuneable drug delivery, tumour hyperthermia and antibacterial behaviour, where gallium addition has been demonstrated in the latter. Specifically, combining LM with nanomaterials or polymers, and in coatings and films, demonstrates outstanding antimicrobial properties in orthopaedic applications. For example, novel composite coatings of hydroxyapatite (HAp) and gallium liquid metal (LM) using atmospheric plasma spray (APS) presented excellent antibacterial efficacies against both initial attachments and established biofilms generated from methicillin-resistant *S. aureus* and *P. aeruginosa* after 18 h and 7 days of incubation in comparison to the control HAp coating [72]. In the work of Li et al. [73], Ga^3+^ released from eutectic gallium-indium alloys (EGaIn) was found to trigger the formation of reactive oxygen species, resulting in bacterial cell death [73], as shown in Figure 7. Furthermore, LM nanodroplets were found to have strong adhesion on bacterial and fungal cell surfaces, leading to membrane disruption [74]. The same research group further developed LM textiles to inhibit the growth of both pathogenic *S. aureus* and *P. aeruginosa* [75].

**Figure 8:**
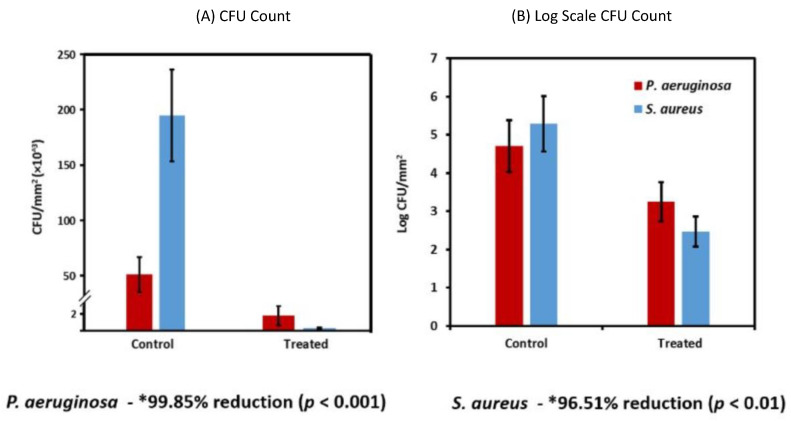
Control and magnetically activated (treated) gallium-based LM effect on *P. aeruginosa* and *S. aureus*: (**A**) Raw colony-forming unit (CFU), and (**B**) logarithmic CFU depiction [76].

This research group [76] also showed the antibacterial and antibiofilm activity of magneto-responsive gallium-based LM droplets after magnetic activation. Assessment against both Gram-negative and Gram-positive bacterial biofilms revealed that more than 99% of bacteria became nonviable after 90 min, where biofilms were permanently destroyed (Figure 8). Another interesting development included that gallium-copper particles were found to outperform copper nanoparticles in terms of antibacterial, antifungal, and antiviral applications [77]. Similar efficacies have also been demonstrated within LM-polymer composite films, where He et al. [78] prepared an antimicrobial and self-healing sample by combining LM nanodroplets with the polymer polydimethylsiloxane (PDMS). *S. aureus* and *E. coli* bacteria were removed with over 90% success, which was attributed to Ga atom exposure.

### 3.4. Gallium in Bioceramic Systems

As the functionalisation of biomaterials receives greater interest for the modulation of modern health issues, bioceramics, with a focus on calcium phosphate (CaP) materials, represent a leading material within this area. These materials have been the subject of research with a focus on their bone regeneration properties and in facilitating bone cell turnover in medical implant applications, where the ability of antibacterial properties to inhibit biofilm formation has been frequently overlooked [79]. Although there is a scarcity of research into antibacterial CaPs, gallium has been employed within some studies for its antibacterial effect when incorporated within these materials. Kurtjak et al. [80,81] incorporated Ga into hydroxyapatite (HAp) to afford a HAp(Ga) sample. By means of the disc diffusion method, clean inhibition zones against the bacteria *P. aeruginosa* were afforded after 24 h, and were attributed to the antimicrobial action of gallium. Though the (HAp(Ga)(TR)) material obtained using transformation and co-precipitation techniques depicted excellent antibacterial activity, the sample (HAp(Ga)(IE)) produced by the ion exchange method depicted superior inhibition of *P. aeruginosa* bacteria. This difference is mostly attributed to the fast ion release. In a later study [82], however, HAp(Ga) functionalised with gold nanoparticles depicted inhibition against *E. coli*, *S. aureus*, and *S. epidermidis*, while non-functionalised HAp(Ga) demonstrated no antibacterial activity. Indeed, recent research has demonstrated the antibacterial activity of Ga incorporated CaPs, but their cytotoxicity remains questionable. Using the same methods, [80] and [81] used human and animal fibroblasts to evaluate the cytotoxicity of HAp(Ga) in vitro. At concentrations of HAp(Ga) less than or equal to the minimal inhibition concentration (MIC), cell viability was reported at approximately 80%. However, both studies indicated that once the MIC was exceeded, the viability of human fibroblasts decreased to 50%. This indicates the sensitivity of these compounds and the necessity of monitoring their potential toxic effects on cell viability. This monitoring is essential to optimise their use in promising biocompatible materials.

## 4. Titanium–Gallium-Based Systems

The integration of gallium into titanium-based materials, namely new-generation β-type alloys, has demonstrated desirable antimicrobial activity, biocompatibility, and improved mechanical properties and microstructures. Ti-Ga-based materials, including coatings, nanomaterials, and alloys, are reviewed based on such properties, with a focus on their suitability for orthopaedic implant applications. The materials that have incorporate gallium coatings onto titanium substrates as reported in the literature are presented in Table 2, with a brief summarisation of their compositions, investigated properties, and notable results. Table 3 includes various Ti-Ga-based alloys found in the literature, and their compositions, properties investigated, and notable results.

### 4.1. Titanium–Gallium Coatings

**Figure 9:**
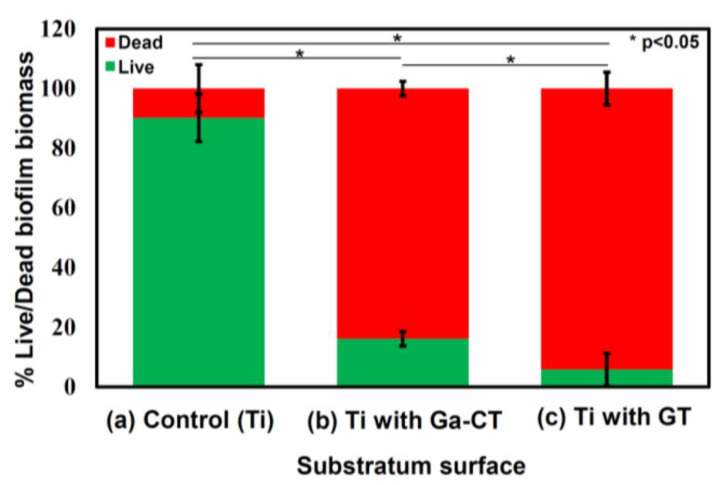
The live/dead biofilm biomass (%) of *A. baumannii* after 7 days from (a) control Ti, (b) Ti with Ga-containing calcium titanate, and (c) Ti with gallium titanate (GT) [85].

The inability of Ti alloys to prevent the onset of bacterial infection after orthopaedic medical device implantation is among the major features affecting their efficacy within biomedical applications. It is clinically imperative to prevent the formation of biofilms, as infections related to biomaterial implantation raise morbidity and mortality rates, which can double following revision surgeries [94]. It has been documented by various studies that Ti alloys doped or alloyed with elements that exhibit an antibacterial activity, such as gallium, depict resistance against biofilm formation and bacterial growth [3,12,18,19,83,85,87,88,89,90,92,94]. For instance, in developing next-generation implant coatings, Stuart et al. [83,84] incorporated gallium into phosphate bioactive glasses (PBG) that were subsequently applied to commercially pure titanium. Antibacterial assays depicted that the Ga-PBG coatings exhibited encouraging results in reducing the viability of *S. aureus* and *E. coli* bacteria, while in vitro cytotoxicity tests on human fibroblast cells indicated good cytocompatibilities. In addition, nano-indentation methods were employed to characterise the mechanical properties of the Ga-PBG coatings, which have only been considered elsewhere in the literature for Ga-coated Ti materials by [19]. For the samples, the elastic moduli were reported within the ranges of 65.3–77.6 GPa, and the hardness values were established within 4.7–7.4 GPa, where increasing gallium addition increased the mechanical properties. With a focus on studying the antibacterial activity and biocompatibility of a similar sample, Yamaguchi et al. [85] explored the antibacterial activity of gallium-incorporated calcium titanate (CT) and gallium titanate (GCT)-coated titanium metal. It highlighted the high antibacterial activity possessed by the material towards *A. baumannii,* and its improved bioactivity (Figure 9). Similarly, other surface coatings have also been applied to titanium implants that were 3D-printed and exhibited comparable results [86].

**Figure 10:**
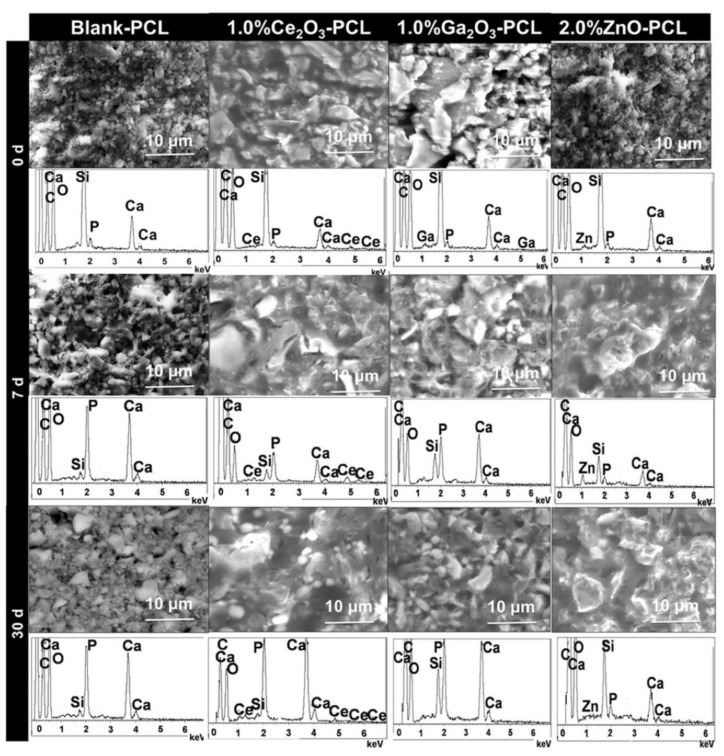
SEM images and EDXS results of blank, cerium, gallium, and zinc glass coatings (MBG-PCL) both prior and following being soaked in simulated bodily fluids. The evolution of the hydroxyapatite surface layer is shown [87].

Using a different approach to develop and assess the prospective bio-functional performance of implant coatings, Shruti et al. [87] employed dip-coating methodologies to deposit gallium-substituted bioglasses onto Ti-6Al-4V alloys before and during simulated bodily fluid treatment. The obtained coatings were homogenous and crack-free, as evidenced by the SEM-EDSX images shown in Figure 10. Furthermore, high in vitro bioactivity was shown by the blank, cerium, and gallium bioglass-coated titanium samples, while the zinc bioglass coating showed low in vitro bioactivity response (Figure 10). However, further investigation into homogenous coating thicknesses is warranted, as this method is known to afford uneven thicknesses across a material. Positive cytocompatibilities were confirmed by in vitro bioactivity assays, and were attributed to gallium addition. By combination of these properties, it is clear that the structural properties of Ti-6Al-4V can be combined with the excellent in vitro bioactivity provided by the incorporation of gallium. Moreover, the use of electrochemical anodization paired with coating techniques can also be considered in the synthesis of gallium-coated materials for implant applications. For example, Dong et al. [88] employed a mixed-methods approach for coating titanium substrate surfaces with Ga^3+^ ions. Initially, electrochemical anodization was used to prepare ordered TiO_2_ nanotubes on the titanium sheets. Samples were then soaked in emulsions, where gallium nitrate bonded to TiO_2_ nanotubes was surface-functionalised to the titanium metal, ultimately allowing for local delivery of gallium ions and therefore the inhibition of *E. coli* and *S. aureus* strains. These strategies of synthesis, paired with antibacterial and cytotoxicity assays, demonstrate the high application potential of gallium-coated titanium materials for orthopaedic implants.

**Figure 11:**
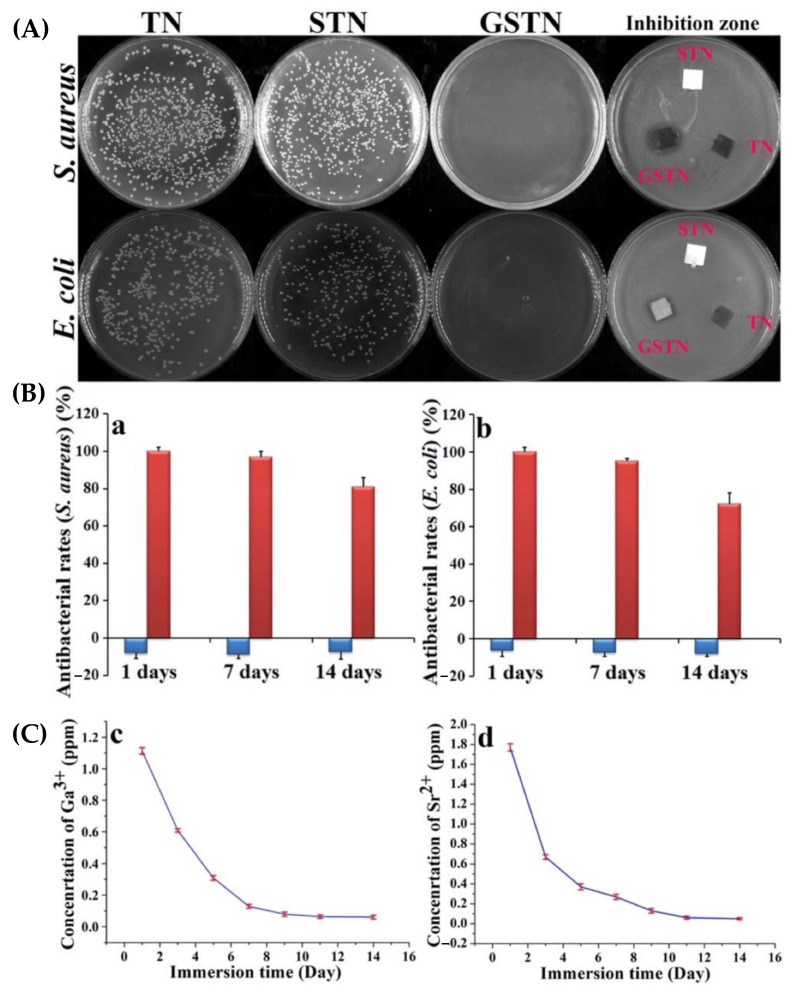
(**A**) Bacteria incubation zones of control (TiO3 nanotubes (TN)), and strontium and gallium from ga-doped TN against cultivated *E. coli* and *S. aureus* after incubation (24 h). (**B**) Antibacterial inhibition rates against (**a**) *E. coli*, (**b**) *S. aureus*, after 1, 7 and 14 days of immersion by a plate-counting method. The blue bar represents the TN sample, and the red bar represents the ga-doped TN sample. Antibacterial ratio (%) = [(N0 − Nt)/N0] × 100%, where N0 = average number of viable colonies (CFU/specimen) for the control sample (TN), and Nt = the average number of the viable bacterial colonies (CFU/specimen) for the test samples. (**C**) Non-cumulative Ga (**c**) and Sr (**d**) profiles of release into PBS from GSNT [90].

To Ti-based implants, Li et al. [89] synthesised a layered double hydroxide (LDH) film composed of strontium and gallium ions using hydrothermal methods. Inhibition of *E. coli* and *S. aureus* was observed, and was due to Ga^3+^’s action against bacterial metabolism. Additionally, using a hydrothermal method, Qiao et al. [90] developed titanium surface-functionalised SrTiO_3_ nanotubes coated with a layer of polydopamine and gallium nitrate. The antibacterial assay depicted that gallium-coated samples prevented bacterial colony formation of *S. aureus* and *E. coli,* and exhibited enhanced antimicrobial action. Notably, after 24 h, almost no bacteria remained, where the substrates did not exhibit a reduction in antibacterial activity until after 7 days, where 72% of antibacterial action was retained for as much as 14 days, as shown in Figure 11 [90]. Although gallium has definite antibacterial effects, this study highlighted its difficulty in releasing adequate quantities of Ga^3+^ over lasting conditions. This effect is owed to gallium being almost completely hydrolysed under physiological conditions. In assessing the cytocompatibilities of a similar sample to that of [90], Chen et al. [91] fabricated a number of Mg-Ga LDH nanosheets on alkali-heat-treated titanium implants. The Mg/Ga-coated titanium implants exhibited promoted osteogenesis, i.e., new bone formation, in addition to suppressed osteoclast generation, thereby indicating superior cytocompatibilities.

**Figure 12:**
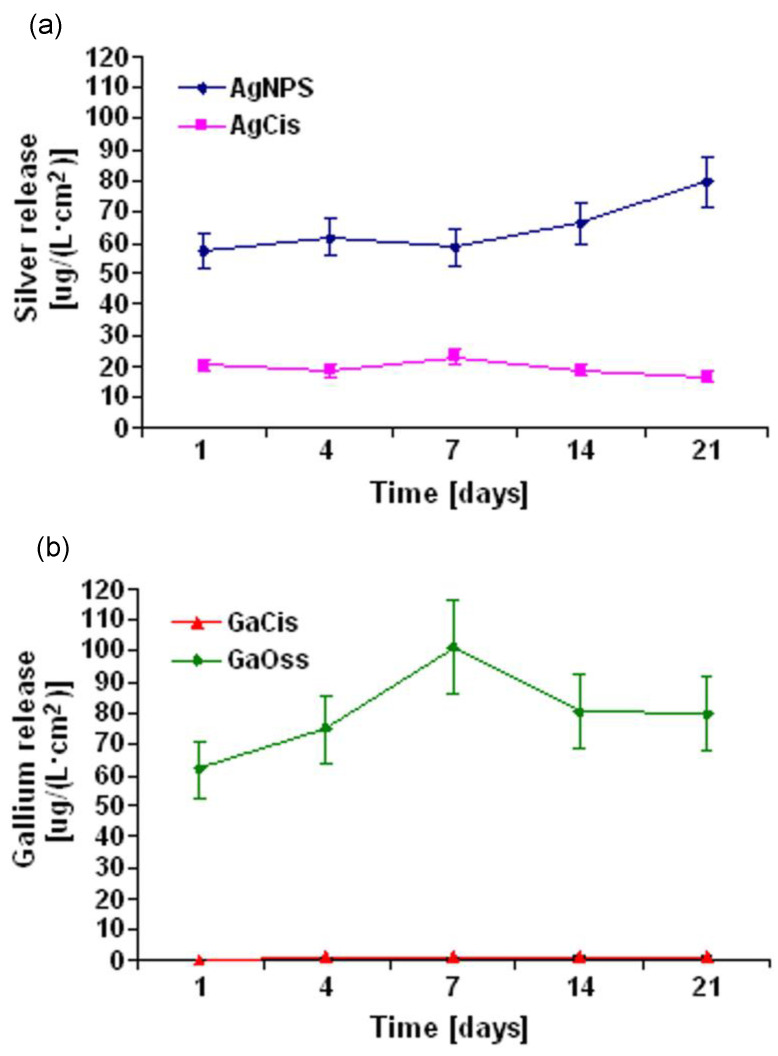
Release of antibacterial agents against A. baumannii measured over 21 days (**a**) Silver release from silver with chelating agent l-Cysteine (AgCis), and silver nanoparticles (AgNPS); (**b**) gallium release from Ga(NO_3_)_3_ with chelating agent l-Cysteine (GaCis), and Ga(NO_3_)_3_ with chelating agent oxalic acid (GaOss) [18].

With a focus on improving the performance of dental implants and other biomedical materials, Cochis et al. [92] investigated the efficacy of gallium-coated titanium surfaces using the anodic spark plasma (ASD) surface modification technique. In vivo antibacterial assays on the dental implant revealed the efficacy of the gallium-coated sample compared to the silver-coated sample, where a strong inhibition of 27–35% against bacterial activity was observed [92]. In a later study by Cochis et al. [18], the authors further investigated the effectiveness of the same samples against multidrug-resistant *A. baumannii*. Again, the Ga-doped Ti sample exhibited stronger bacterial inhibition against various strains of *A. baumannii* compared to that of Ag-doped Ti, in addition to exhibiting no cytotoxic effect [18]. Gallium therefore imparted a crucial role in discouraging the colonisation and growth of *A. baumannii* on the surface of the implant due to its gradual release from the coating. The gradual release kinetics of gallium is shown in Figure 12, where the GaCis sample depicted a minor and gradual release of gallium (Figure 12b) from the fourth day (1.1 μg/(Lcm^2^)), from which it was then stable until day 21. Importantly, no detrimental impact to the mechanical properties was observed with the addition of gallium to the titanium scaffolds. However, the evaluation of mechanical properties did not receive extensive analysis as only the elastic modulus, hardness, critical load, and shear stress properties were assessed using nano-indentation methods. Though it was revealed that the hardness exceeded that of the titanium alloy control, other properties did not differ significantly from the titanium control. Further research is therefore warranted to comprehensively elucidate the effect of gallium on the mechanical properties of titanium alloy systems. Overall, the collective findings suggest that gallium-coated titanium surfaces possess potent antibacterial activity in improving the performance of dental implants and within the field of antibacterial surface modifications.

### 4.2. Titanium–Gallium-Based Alloys

**Figure 13:**
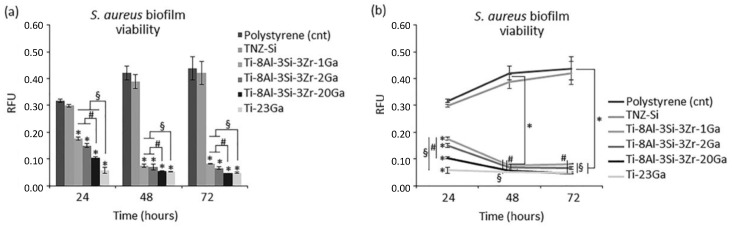
Antibacterial effect of the Ti-8Al-3Si-3Zr-(1, 2, 20 wt%)-Ga alloys and control samples: (**a**) metabolic activity at 24, 48, 72 h of *S. aureus*; (**b**) metabolic activity over time of *S. aureus*. Values are expressed as means and standard deviations; all experiments were performed in triplicate, *p* < 0.05. * indicates *p* < 0.05 for Ga-doped samples compared to the polystyrene control, and # and § indicate that 20Ga and 23Ga samples observed *p* < 0.05 compared to other Ga-based samples, respectively [19].

**Figure 14:**
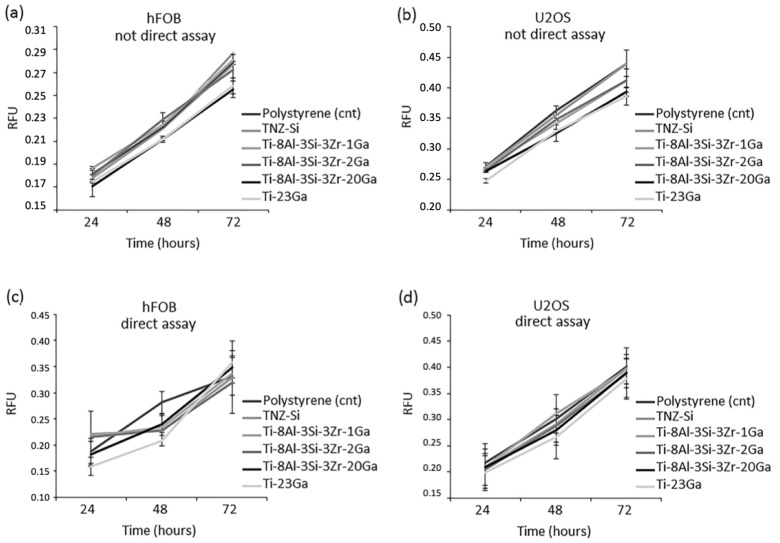
Cytocompatibility assessment of the developed alloy systems (Ti-8Al-3Si-3Zr-XGa (X = 1, 2, 20)) and control samples. (**a**,**b**) Indirect assay using hFOB (**a**) and U2OS (**b**) cells. (**c**,**d**) Direct assay using hFOB (**c**) and U2OS (**d**) cells [19].

Metal alloys are frequently employed as clinical orthopaedic implants, where the addition of alloying elements such as gallium can prevent biofilm formation at the implant surface, and show bactericidal activity. Cochis et al. [19] metallurgically added gallium (1, 2, 20 wt%) to the titanium alloy Ti-Al-Zr-Si and reported the successful inhibition of *S. aureus* bacteria, with more than an 80% reduction in the metabolic activity of the bacterial strain compared to the control sample, as shown in Figure 13. Promisingly, even 1–2 wt% additions of Ga to Ti-Al-Zr-Si alloys depicted potent antibacterial efficiency [19]. Although it was demonstrated that the samples ensured the release of Ga^3+^ ions and showed strong antibacterial effects, this efficacy was only observed for at least 3 days. However, the authors expect that over longer study periods, the antibacterial activity would exhibit the same trends [19]. In addition to the antibacterial effect, the cytocompatibility was analysed by both direct and indirect assays with mature osteoblast and preosteoblast cells, in which great cytocompatibility was revealed (Figure 14). Though mechanical properties were not investigated, it is predicted that greater concentrations of gallium additions would induce the mechanical properties, as gallium is an alpha-phase stabiliser and should therefore be kept low when alloyed [19].

**Figure 15:**
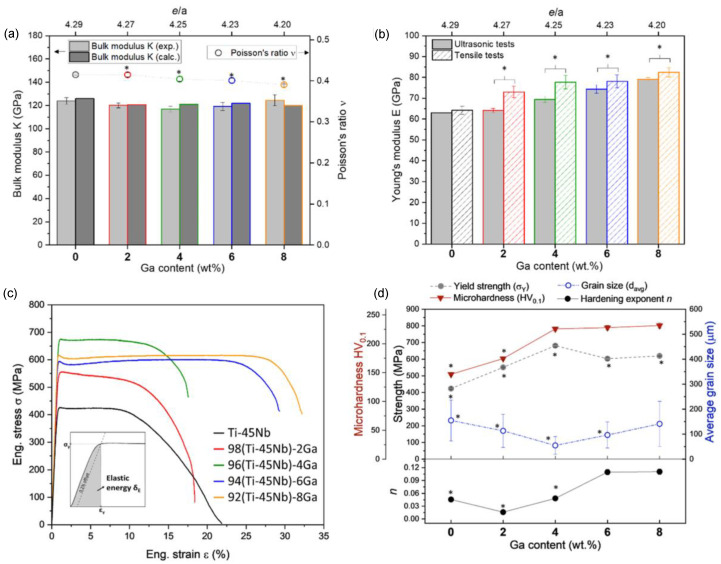
(**a**) Poisson’s ratio (v) and Bulk modulus (K) plotted with increasing Ga additions (wt%). (**b**) Young’s modulus assessed experimentally (tensile tests) compared to the ultrasonic pulse-echo method. (**c**) Engineering stress–strain curve depicting yield strength and yield strain. (**d**) Yield strength, grain size, and Vickers microhardness plotted with increasing Ga addition (wt%). The most important comparisons are labelled (*) [12].

Indeed, alloying of Ga to Ti-Nb alloys and its effect on transformation temperature properties have been investigated, but its influence on mechanical behaviour has not been investigated until the work of Alberta et al. [12,93,95]. In investigating Ti-45Nb-xGa (x = 2, 4, 6, 8 wt%) alloys, it was revealed that the master alloy paired with 4 wt% Ga depicted the best mechanical properties. This included a near 40% increase in strength over Ti-45Nb [12]. Through tensile and microhardness tests, increasing additions of gallium were found to improve the strength of the alloys, with the maximum yield strength and microhardness being 620 ± 2 MPa and 232 ± 5 HV, respectively (Figure 15) [12]. The same trends were observed for the Young’s modulus and ductility, with values in the range of 73 ÷ 82 GPa for stiffness, and a maximum ductility of 32% (Figure 15) [12]. Therefore, the addition of gallium to Ti-45Nb alloys affords a desirable balance between a low Young’s modulus and increased strength.

**Figure 16:**
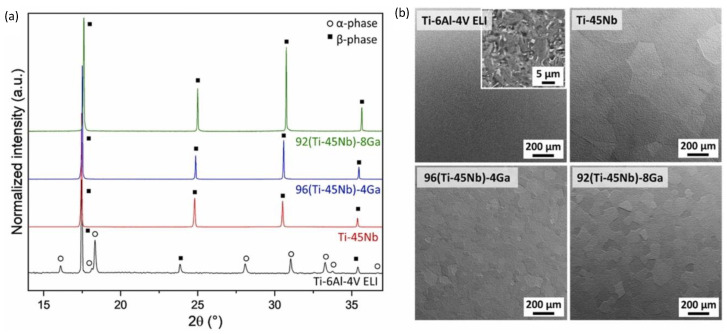
(**a**) XRD patterns and (**b**) SEM images of the Ti-45Nb-(4, 8)Ga alloys compared to Ti-6Al-4V [95].

In another study, the same authors also investigated the influence of both Ga and Cu on the corrosion characteristics, phase constitution, and mechanical properties when alloyed to Ti-45Nb [95]. Using simulated body fluids to assess the corrosion characteristics, no deleterious effect on the corrosion resistance was observed with the addition of Ga. By means of X-ray diffraction techniques and microstructural analysis, all alloys depicted a single β-phase structure, which is desirable for orthopaedic implants, as shown in Figure 16. Excellent plasticity was detected and attributed to work hardening, where similar tensile strengths and Young’s modulus results were found compared with those of the initial study. Overall, each study emphasised that the addition of Ga gave a strengthening effect to the alloy, while retaining a low Young’s modulus. This is believed to be due to the grain refinement effect shown in SEM images in Figure 16b; grain refinement is well reported to increase hardness properties. It was clarified that Ga addition should be kept low to prevent the β-phase transforming to α or α-β type phases, where the 4 wt% addition exhibited the best combination of mechanical properties and microstructure. However, the antibacterial effect of gallium’s addition to Ti-Nb alloys has not been studied, nor has its cytotoxicity. This demonstrates the need to investigate the effect of Ga addition on Ti-Nb alloys in terms of its antibacterial and cytocompatibility effects, paired with determination of their mechanical and microstructural properties.

## 5. Suggestions for Future Research

Although exciting findings have been reported in the development of gallium material systems, especially for Ti-Ga-based materials, further research is warranted to explore the comprehensive microstructural, mechanical, antibacterial, biocompatible and tribocorrosion properties of numerous compositions. As reviewed in Section 4.1 and Section 4.2, very little work has encompassed investigation into the mechanical properties of gallium–titanium material systems. In addition, although Ti-Nb alloys have emerged as the focus of research into improved biomaterials, there is scarce research into Ti-Nb-Ga based alloys. Given the promising antibacterial properties of gallium reviewed in Section 3 and Section 4, it is suggested that future research focuses on elucidating the optimal gallium concentration when added to Ti-alloys, and subsequent comprehensive studies on the mechanical, microstructural, and biocompatible properties of the materials. Research into their corrosion resistance and deeper work on their osteointegration and osteogenic differentiation are also warranted to assess their suitability for use in biomedical applications. Furthermore, the mechanism of gallium release should be elucidated to gain a deeper understanding of its release kinetics, particularly within permanent material systems such as Ti-Ga alloys.

Future work could also be directed towards studying advanced manufacturing techniques and their effect on the mechanical and biocompatibility properties of titanium alloys. As gallium has been shown to stabilise the α-phase, this could be advantageous in improving mechanical properties while still imparting antimicrobial activity. The emergence of nanomaterials in recent years has allowed for materials to be endowed with excellent antibacterial properties. Further research into this development could allow for their combined use with gallium to produce promising biomedical materials that exhibit optimal antibacterial activity.

## 6. Summary

New-generation titanium alloys with biocompatible elements and low stiffness levels represent a promising class of materials for use in biomedical implant applications, particularly orthopaedic implants. The addition of alloying elements, such as gallium, can impart advantageous properties including antibacterial activity and increased biocompatibility. Gallium has been applied to various materials, including bioglasses, liquid metals, bioceramics, and titanium-based materials and alloys, and has continually demonstrated desirable antimicrobial behaviour against multiple Gram-negative and Gram-positive bacterial strains. When alloyed with β-type Ti-alloys, such as Ti-Nb-based alloys, its impressive ability to inhibit bacteria makes it a promising material in mitigating the risk of implant-associated infections and in improving patient outcomes within orthopaedic implant applications. If a balance can be achieved between mechanical properties, antibacterial efficiency and cytocompatibility, gallium-containing titanium alloys can reduce the incidence of implant failure and enhance the biocompatibility and overall performance of medical implants. However, future research should be directed towards further elucidating their synergistic biological and mechanical properties, as this is largely deficient in the literature.

## Figures and Tables

**Table 1:** Assessed biocompatibility and biological impacts of various chemical elements, some of which can be incorporated into titanium alloy systems; red, yellow and green indicate serious, moderate, and minimal concerns, respectively. Other* pertains to concerns that extend beyond those previously mentioned. Examples include haemolysis, neurological effects, and so forth [27].

Periodic Position	Element	Biocompatible	Carcinogenic	Genotoxic	Mutagenic	Cytotoxic	Allyergenic	Prone to Corrosion	Other*
3d	Ti	Yes	No	No	No	Med	No	No	No
	V	No	Yes	Yes	Yes	High	Disputed	No	No
	Cr	No	Disputed	Yes	Yes	High	Yes	No	No
	Mn	No	No	Yes	No	High	No	Yes	No
	Fe	No	No	Yes	Disputed	Med	No	Yes	No
	Co	No	Yes	Yes	Yes	High	Yes	Yes	Yes
	Ni	No	Yes	Yes	Yes	High	Yes	Yes	Yes
	Cu	No	No	Yes	Yes	High	Yes	Yes	Yes
4d	Zr	Yes	No	No	No	Low	No	No	No
	Nb	Yes	No	No	No	Low	No	No	No
	Mo	No	Disputed	Yes	Yes	Low	Yes	Yes	Yes
	Tc	No	-Radioactive-						
	Ru	Yes	No	No	No	Med	No	No	Yes
	Rh	No	Yes	Yes	Yes	High	Unknown	No	No
	Pd	No	Yes	No	Disputed	Med	Yes	No	No
	Ag	No	No	No	No	High	Yes	No	Yes
5d	Hf	Unknown	Unknown	Unknown	Unknown	Med	No	No	Unknown
	Ta	Yes	No	No	No	Low	No	No	No
	W	No	Yes	Yes	No	Med	No	Yes	No
	Re	Unknown	Unknown	Unknown	Unknown	Unknown	No	No	Unknown
	Os	No	Unknown	Yes	Yes	High	No	Yes	No
	Ir	No	No	No	Yes	High	No	No	Yes
	Pt	No	Yes	Yes	Yes	High	Yes	No	No
	Au	Yes	No	No	No	High	No	No	No
Other	Al	No	No	Yes	No	Low	No	No	Yes
	Zn	No	No	No	No	High	No	No	Yes
	Sn	Yes	No	No	No	Low	No	No	Yes

**Table 2:** Gallium coatings applied to titanium substrates and their results, as reported in the literature.

Substrate	Coating(s)	Properties Investigated/Methods	Notable Results	Ref.
Commercially pure Ti	P_2_O_5_-CaO-MgO-Na_2_O-XGa_2_O_3_ (X = 6, 8.6 mol%).	Elemental mapping and microstructural analysis. Surface roughness and surface features. Mechanical properties (reduced moduli, elastic modulus, hardness). Cytocompatibility tests. Antibacterial assays.	All samples cytocompatible. Antibacterial activity effective at 24 h for Gram-positive and Gram-negative bacteria.	[83,84]
Ti	GaCl_3_	Surface morphology. Ion release. Antibacterial activity.	Notable antibacterial activity against *A. baumannii.* Up to 94.2% biofilm removal.	[85]
Porous Ti	Ga(NO_3_)_3_	Surface characterisation. Ion release. Antibacterial assay. Cytotoxicity and biocompatibility assays.	Effectively inhibited *P. aeruginosa.* Effective osteogenic differentiation and mineralisation on Saos-2 cells.	[86]
Ti-6Al-4V	Mesoporous bioactive glass substituted with Ce, Ga, Zn.	Surface characterisation. Cytotoxicity and biocompatibility assays.	Homogenous and crack-free coatings. Positive cytocompatibilities.	[87]
TiO_2_ nanotubes Pure Ti sheets	Ga(NO_3_)_3_-PDLLA	Biofilm characterisation, cytotoxicity, and biocompatibility assays. Antibacterial assay.	Inhibition of *E. coli* and *S. aureus* bacteria.	[88]
Ti	LDH-Ga and Sr	Biofilm characterisation, cytotoxicity, and biocompatibility assays. Antibacterial assay.	Enhanced differentiation of cells and osteoblasts. Antimicrobial inhibition against *E. coli* and *S. aureus.*	[89]
SrTiO_3_ nanotubes on Ti	Ga(NO_3_)_3_-PDA	Surface characterisation. Antibacterial assay. Cytotoxicity and biocompatibility assays.	Superior osteoinductive activity. Gradual and constant antibacterial agent release of *E. coli* and *S. aureus*. Almost no bacteria after 7 days.	[90]
Ti	Ga(NO_3_)_3_	Surface characterisation. Cytotoxicity, osteogenesis and osteoclastic biocompatibility assays.	Promoted osteogenesis, suppressed osteoclast generation.	[91]
Grade 2 Ti	GaCis and GaOss (Ga(NO_3_)_3_)	Morphological characterisation. Mechanical properties (elastic modulus, hardness). Antibacterial assay. Cytotoxicity and biocompatibility assays.	Strong inhibition of bacteria between 27–35%. Inhibition of *A. baumannii.* Good cytocompatibilities.	[18,92]

**Table 3:** Metallurgic addition of gallium to titanium, including key methods and results.

Chemical Composition (wt%)	Properties Investigated/Methods	Notable Results	Ref.
Ti-8Al-3Si-3Zr-1Ga Ti-8Al-3Si-3Zr-2Ga Ti-8Al-3Si-3Zr-20Ga	Microstructural analysis. Antibacterial assays. Biocompatibility/cytotoxicity assays.	Inhibition of *S. aureus*, more than 80% reduction in metabolic activity. Potent antibacterial efficiency for all samples, even 1–2 wt% additions of Ga. Great cytocompatibilities.	[19]
Ti-45Nb-2Ga Ti-45Nb-4Ga Ti-45Nb-6Ga Ti-45Nb-8Ga	Chemical composition analysis. Mechanical properties (yield strength, Young’s modulus, hardness, ductility).	4 wt% Ga depicted the best combination of mechanical properties. 40% increase in strength over Ti-45Nb. Maximum yield strength: 620 ± 2 MPa. Microhardness: 232 ± 5 HV. Young’s modulus: 73 ÷ 82 GPa. Maximum ductility: 32%	[12,93]
Ti-45Nb-4Ga Ti-45Nb-8Ga	Microstructural analysis. Mechanical properties. Corrosion and tribocorrosion properties.	Single β phase. Ga caused no deleterious effect on the corrosion resistance.	[93]

## Data Availability

No new data were created.
